# The Clinical Significance of Tonsillar *Actinomyces* in Histopathological Samples after Tonsillectomy

**DOI:** 10.3390/pathogens12121384

**Published:** 2023-11-24

**Authors:** Natalia Zięba, Katarzyna Miśkiewicz-Orczyk, Maciej Misiołek, Wojciech Ścierski

**Affiliations:** Department of Otorhinolaryngology and Laryngological Oncology in Zabrze, Medical University of Silesia, st. Marii Curie-Skłodowskiej 10, 41-800 Zabrze, Polandmaciej.misiolek@sum.edu.pl (M.M.); wojscier@mp.pl (W.Ś.)

**Keywords:** *Actinomyces*, tonsillectomy, obstructive sleep apnea, tonsillitis, oral microbiome, palatine tonsil

## Abstract

Background: *Actinomyces* is a genus of Gram-positive anaerobic or microaerophilic bacteria with a branched filamentous shape. Their presence in tonsil tissue is usually determined by histopathological examination. In the crypts of removed tonsils, they have a prevalence ranging from 0.8% to 61.6%. The role of *Actinomyces* in diseases of the palatine tonsils is not clearly defined. The aim of the study was to determine the importance of *Actinomyces* in the pathology of palatine tonsils and to assess the impact of these bacteria on the clinical data. Methods: the retrospective analysis of the histopathological findings of patients undergoing tonsillectomy in our hospital from January 2017 to the end of December 2019 was performed in terms of the occurrence of *Actinomyces*. The data were collected based on the medical history. The study included 481 patients aged 3–82 years. From the study group, 100 adult patients were randomly selected, and a telephone survey was conducted. The questions included the co-occurrence of bronchial asthma, halitosis, tonsilloliths, and cigarette smoking. The questions were related to the complications following tonsillectomy and the severity of postoperative pain. Existence of a relationship was investigated between occurrence of tonsillar *Actinomyces* and age, sex, body mass index, and medical condition (obstructive sleep apnea, chronic palatine tonsillitis), respectively. The size of the removed tonsils was assessed and compared depending on the presence of the bacteria. Results: patients aged 18 years and older had a higher probability of presenting *Actinomyces*. The estimated odds ratio for the presence of the bacteria per year of age was 1.023 [1.007, 1.041]. No statistically significant results were found for the other variables. The co-occurrence of the bacteria and halitosis was close to statistical significance (*p* = 0.064). Conclusions: multivariate analysis of the role of *Actinomyces* in tonsillar pathology showed that these microorganisms should be considered saprophytes of the oropharyngeal microflora that had no significant relationship with the pathology of palatine tonsils. Further studies on their influence on halitosis are warranted.

## 1. Introduction

*Actinomyces* is a genus of Gram-positive, slow-growing, non-spore-forming, and non-acid-resistant anaerobic bacteria with a branched filamentous shape [1,2,3,4,5]. *Actinomyces* is a part of the physiological flora of the oral cavity, throat, intestines, genital tract, and skin in humans [5,6,7]. However, these bacteria can sometimes be an etiological factor in the development of infections [5]. They can penetrate soft tissues, leading to infection which is favored by hypoxia associated with inflammation resulting from trauma, surgery, cancer, or another infectious disease [2,7,8,9,10]. Actinomycosis can involve all tissues and organs [11]. The disease process is most often related to the cervicofacial region (55%), the abdominopelvic region (25%), and the pleural-thoracic region (15%), with 5% of cases involving another location [2,9,12,13,14]. The symptoms of actinomycosis are site-specific. The most predominant clinical features include abscesses, poorly healing fistulas, nodular lesions, and granulomatous fibrous inflammatory infiltrates. In the most common cervicofacial manifestation, the disease process primarily involves the soft tissues and the mandibular region, and less frequently affects the oral cavity, tongue, or pharynx. In all cases, actinomycosis should be differentiated from a neoplastic process [13].

**Figure 1:**
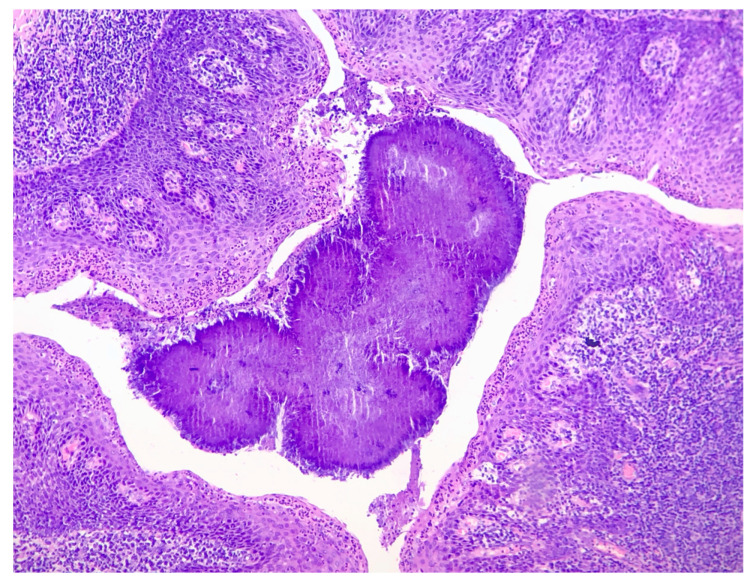
A histopathological sample of the palatine tonsil at 100× magnification subjected to hematoxylin and eosin staining. The *Actinomyces* colonies can be seen in the crypt of the tonsil, covered by nonkeratinizing squamous epithelium.

*Actinomyces israeli* is the most commonly isolated species of *Actinomyces* [2,5,6,11,15]. In the oral cavity, *Actinomyces* has been found in carious dentin, dental plaque, on the gingival surface, and in tonsillar crypts [5,16,17,18]. The presence of tonsillar *Actinomyces* is confirmed by the assessment of histopathological samples of filamentous clusters of basophilic microorganisms arranged in a radial pattern (“ray-fungus”) or bacterial cultures [4,5,19]. Hematoxylin and eosin staining is considered an effective method for identifying these bacteria (Figure 1) [1,14].

According to the literature, reports on the role of *Actinomyces* in palatine tonsil diseases are inconclusive [1,2,3,4,18,19,20,21,22,23,24]. Some studies have found that they are commensals, fortuitously detected during postoperative histopathological examination, and their presence is not indicative of active disease. Other studies stressed their significant role in the development of tonsillar pathology. Treatment of some conditions could primarily rely on pharmacotherapy, thus reducing the frequency of tonsillectomies associated with elevated postoperative pain and the risk of bleeding. Additionally, if proven, an association between *Actinomyces* bacteria and the occurrence of postoperative complications, along with increased postoperative pain, could lead to the implementation of appropriate perioperative antibiotic prophylaxis to decrease the incidence of these complications. The disparities in research findings and the potential benefits of determining the role of *Actinomyces* were the reasons for conducting this study.

The primary aim of the study was to determine the importance of bacteria of the genus *Actinomyces* in the pathology of the palatine tonsils, such as chronic tonsillitis, hypertrophy, asymmetry, and formation of tonsilloliths. In addition, an attempt was made to determine whether the presence of *Actinomyces* could be associated with obstructive sleep apnea syndrome (OSAS) and halitosis.

## 2. Materials and Methods

The study was conducted between 2019 and 2022. It consisted of a retrospective component and a survey. The study group comprised 481 patients (aged 3 to 82 years) who underwent surgery in the Department of Otorhinolaryngology and Laryngological Oncology in Zabrze, Medical University of Silesia, Katowice from January 2017 to the end of December 2019. All patients underwent bilateral tonsillectomy due to chronic tonsillitis (*n* = 405) or OSAS (*n* = 76). The study included 89 children and 392 adults (205 women, 276 men). A retrospective analysis of data on the study group was performed and a telephone survey was conducted among a randomly selected group of 100 patients.

The inclusion criteria were as follows: bilateral tonsillectomy due to chronic tonsillitis or OSAS and availability of histopathological results in the medical records. The exclusion criteria were as follows: lack of informed written consent to participate in the study, lack of histopathological findings in the medical history, and tonsillectomy due to oncological indications.

The palatine tonsils were removed by different surgeons, always following the same steps performing the cold steel tonsillectomy. Next, the samples in formalin were transferred to the histopathology laboratory. Histological samples were collected from the removed tissue. Hematoxylin and eosin were used to stain the histological sections. The presence of *Actinomyces* in the samples was assessed qualitatively. We compared the group of patients who presented the bacteria in the palatine tonsils with the group of patients in whom the bacteria were not found upon histopathological examination. We analyzed the dependence of the presence of tonsillar *Actinomyces* in the specimens on factors such as age, sex, weight, OSAS/chronic tonsillitis, and the variables included in the telephone survey.

The size of the palatine tonsils was given In cm^3^, and the result was obtained by multiplying the length, height, and width of the palatine tonsils. In each patient, the average size of the palatine tonsil from both removed tonsils was taken unless asymmetry was found. The prevalence of tonsillar asymmetry in each group was analyzed according to the presence of *Actinomyces* (a subdivision into the detailed groups is included in the “results”).

The survey section was based on a telephone questionnaire that involved 100 respondents who were randomly selected from the group of adult patients included in the retrospective part of the study. The survey was conducted on a subpopulation of adults due to the previously estimated higher prevalence of *Actinomyces* in this group of patients. The questions were related to the co-occurrence of bronchial asthma, tobacco smoking, halitosis, tonsilloliths, postoperative complications, such as bleeding or infection of the surgical site, and the severity of postoperative pain assessed using the Visual Analog Scale (VAS). 

The limitations of the study were as follows: variables, such as smoking and the presence of *Actinomyces*, were assessed qualitatively, and the presence of halitosis was assessed based on the subjective patient evaluation. Antibiotic therapy in the postoperative period was considered to be due to a surgical site infection. 

All analyses and data transformations were performed using the R statistical environment 4.0.5 [25]. A *p*-value of 0.05 was used as the basis for rejection of the null hypothesis for a given test. Hypotheses were tested using the following procedures: in the case of logistic regressions, the model fit was tested in relation to simpler models (with fewer explanatory variables) using the log-likelihood ratio test. The values of the χ^2^ statistic and the *p*-statistic were reported. Logistic regressions were fitted using the glm() function. In the case of the logistic models with quantitative explanatory variables, tests were performed to assess the fit of the models to the data as suggested by Hosmer, Lemeshow, and Sturdivant using the gof() function (LogisticDX) [26,27]. The ordinal regression model was fitted using the ordinal package [28]. It was assumed that the assigned category of pain was based on unmeasurable pain, which could approximate the normal distribution. Ordinal models were also compared using the log-likelihood ratio test. The comparison of the means was done using the Welch’s *t*-test. Variable independence tests were performed using Pearson’s χ^2^ statistic. 

For statistical purposes, data regarding names and contact details were made confidential. Therefore, patient identification was impossible.

## 3. Results

In the entire study population (*n* = 481), *Actinomyces* occurred in 31.4% of patients. We performed the analyses involving 477 patients to determine the prevalence of the bacteria depending on age, sex, BMI, and disease status. 

First, the probability of the occurrence of *Actinomyces* was estimated based on age (<18 years of age or ≥18 years). The logistic regression model was fitted with age (explanatory variable) and occurrence of *Actinomyces* colonies (explained variable). 

**Figure 2:**
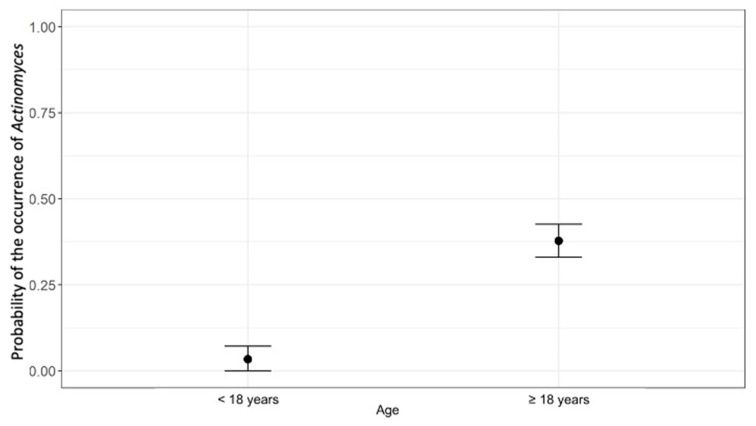
Estimated probability of the occurrence of *Actinomyces* according to age. Dots represent the estimated mean; horizontal lines represent the lower and upper confidence intervals.

*Actinomyces* was significantly more prevalent in patients above 18 years of age (37.8%) compared with those below 18 years of age (3.4%). The model with the explanatory variable was significantly better than the null model, in which the explanatory variable was not included (χ^2^ = 51.98, *p* < 0.001). The effect was statistically significant. Patients aged 18 and older had a higher probability of presenting *Actinomyces*. The estimated probabilities are shown in Figure 2.

Eighty-eight patients younger than 18 years of age at the time of the study were excluded from further analysis. Table 1 shows descriptive statistics of age for patients with and without *Actinomyces*.

*Actinomyces* was more prevalent in men than women (32.2% vs. 30.20%). In patients with OSAS, *Actinomyces* was found in 43.4% of study subjects and in 29.1% of patients with chronic palatine tonsillitis. After excluding children from the analysis, the prevalence of *Actinomyces* was 43% (OSAS group) compared to 36.6% (chronic tonsillitis group).

**Figure 3:**
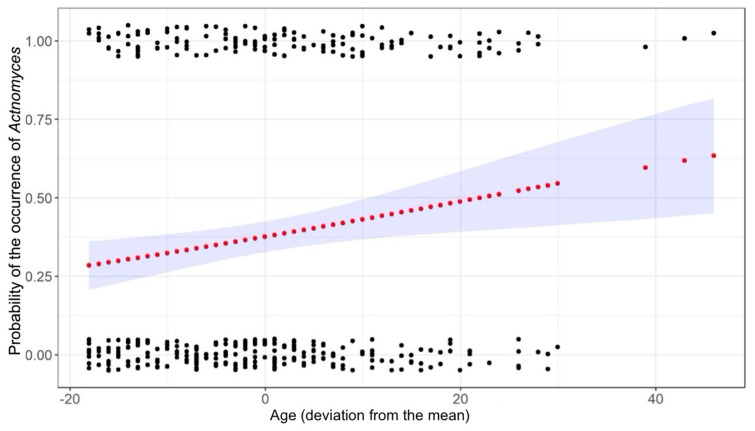
Probability of the occurrence of *Actinomyces* according to age. black dots represent the observed data, red dots represent the predicted data, the blue area represents a 95% confidence interval around the estimates.

The regression model fitting proposed by Hosmer, Lemeshow, and Sturdivant was applied. In the first step, we checked which explanatory variables influenced the occurrence of *Actinomyces* in individual models. Variables for which *p* < 0.2 were considered potentially influential. The result of Pearson’s χ^2^ test was χ^2^ = 0.002 (*p* = 0.963) for sex and χ^2^ = 1.156 (*p* = 0.282) for disease status. However, the differences were not statistically significant. Next, the model was tested with respect to age and categorized BMI, the latter of which was found to be statistically non-significant (χ^2^ = 1.85, *p* = 0.397). A statistically significant effect of age on the prevalence of *Actinomyces* was determined. The estimated odds ratio for the occurrence of *Actinomyces* per year of age was 1.023 [1.007, 1.041]. Figure 3 shows the estimated probability of the occurrence of *Actinomyces* according to age.

Further variables were tested using logistic regression models. Smoking had no effect on the presence of *Actinomyces* in tonsils (χ^2^ = 0.01, *p* = 0.922, *n* = 145). The estimated probability of postoperative bleeding was not significantly associated with the presence of *Actinomyces* in the histopathological samples (χ^2^ = 0.08, *p* = 0.778, *n* = 100). Complications in the form of surgical site infection were not related to the presence of the bacteria in the postoperative samples (χ^2^ = 0.55, *p* = 0.458, *n* = 98). No significant relationship was found between the co-occurrence of bronchial asthma and tonsillar *Actinomyces* (χ^2^ = 0.42, *p* = 0.516, *n* = 118).

**Figure 4:**
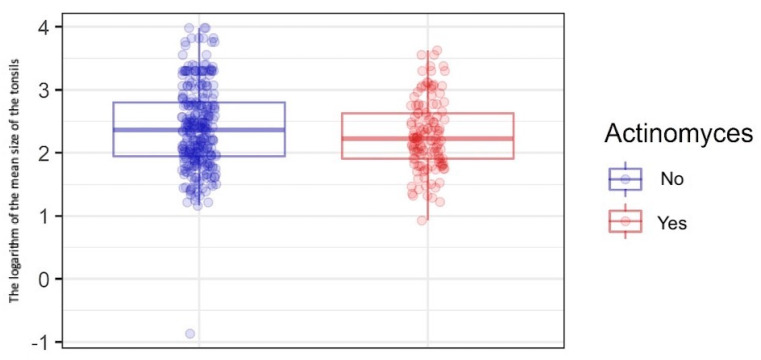
Distribution of the logarithmic variable (mean tonsil size) according to the groups. The horizontal line in the center of the two rectangles represent the median, the lower and upper edges represent the first and third quartiles, the vertical lines represent the values of 1.5 × interquartile range, and the dots represent observations.

To determine the effect of *Actinomyces* on tonsillar size and the occurrence of tonsillar asymmetry, the comparison of the means of the size of tonsils was performed using Welch’s t-test depending on the presence of *Actinomyces*. The analysis was performed in 419 patients for whom data regarding the tonsil size were available. Due to the right-skewed distribution of the variable according to the groups, a logarithmic transformation was performed before the analysis. The presence of *Actinomyces* in the palatine tonsil samples was not associated with tonsillar hypertrophy. The mean size of tonsils was smaller in the group of patients with *Actinomyces*. Logarithmic mean tonsil size was 2.27 for patients with *Actinomyces* and 2.39 for those without *Actinomyces* (Table 2 and Figure 4).

We calculated the absolute value from the difference between the sizes of the tonsils to determine the effect of *Actinomyces* on the occurrence of asymmetry. The variable was divided into four approximately equal intervals, which can be interpreted as follows:

1. No or negligible asymmetry (0–0.5);
2. Slight asymmetry (0.5–2.25);
3. Moderate asymmetry (2.25–5.07);
4. High asymmetry (>5.07).

**Figure 5:**
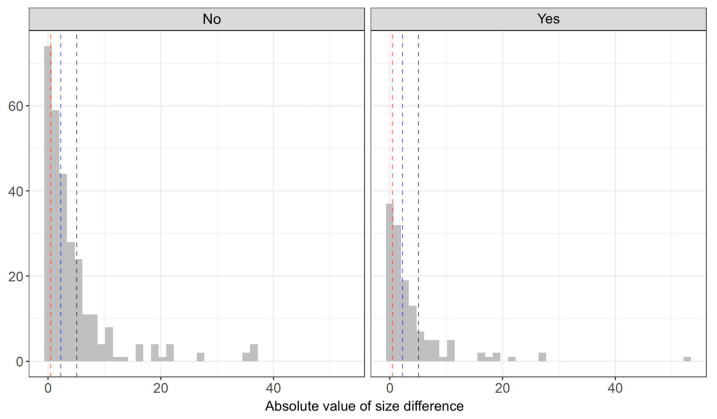
Distribution of tonsillar asymmetry according to the occurrence of *Actinomyces*.

Figure 5 shows the distribution of all values of the variable according to groups with the edges of the intervals.

The test of independence of variables was performed using Pearson’s χ^2^ statistic. However, the results were not statistically significant. Therefore, the null hypothesis of independence of the variables could not be rejected. Based on the data obtained from 100 patients participating in the survey, no significant relationship was observed between the presence of tonsilloliths and *Actinomyces* colonies (χ^2^ = 0.01, *p* = 1). No statistically significant association was found between halitosis and the presence of *Actinomyces* colonies in tonsil samples (χ^2^ = 3.66, *p* = 0.064).

The ordinal regression model was fitted to the data to assess the effect of *Actinomyces* on the severity of postoperative pain. The analysis involved 99 patients who provided information on the severity of postoperative pain. The highest number of respondents (45.5%) reported experiencing severe pain after tonsillectomy (VAS 7–9), 14.1% described the pain as unbearable (VAS 10), and only 7.1% reported mild pain (VAS 1–3). The remaining respondents described the severity of pain as moderate (VAS 4–6). No significant differences were noted in the severity of pain after palatine tonsillectomy depending on the presence of *Actinomyces* in the postoperative samples (χ^2^ = 0.245, *p* = 0.117).

## 4. Discussion

The influence of *Actinomyces* on the occurrence of pathological processes in the palatine tonsils has been analyzed for many years [1,2,3,4,8,18,19,20,21,22,23,24]. Confirmation of the relationship between the presence of tonsillar *Actinomyces* and the prevalence of postoperative bleeding, increased postoperative pain, or the need for postoperative antibiotic therapy associated with surgical site infection could allow the implementation of appropriate prophylactic measures. Demonstrating a positive correlation between palatine tonsillar hypertrophy and the occurrence of *Actinomyces* could change the approach to the treatment of patients whose main problem is related to obstruction of the upper respiratory tract associated with hypertrophy of the pharyngeal lymphoid tissue. 

In our study, *Actinomyces* was found in 31.4% of patients. In the literature, the prevalence of *Actinomyces* colonies in palatine tonsils removed due to recurrent tonsilitis and OSAS ranges from 0.8% to 61.6% [2,14,16,29,30,31,32]. In their study on a large cohort of patients, Aydin et al. found that this percentage was 6.7% for the entire study population, including children (*n* = 1252) and adults (*n* = 568) [31]. On the other hand, Toh et al. obtained results similar to our findings. They examined 834 patients (72 patients aged 1–16 years, 762 patients aged 17–68 years), with *Actinomyces* found in 35.6% of samples [17]. This disproportion may be due to the fact that Aydin et al. assessed a significant group of children, while in the study of Toh et al. adults were predominant. Similarly, the adult population was predominant in our study. The analyzed data included 391 adults and 89 children. The mean age of the study population was 30.82 ± 15.7 years. The study showed a statistically significant effect of age on the prevalence of *Actinomyces*, which is in line with many study findings [2,4,14,19,24,30,32]. Only Gaffney et al. obtained results that indicated that *Actinomyces* was more prevalent in patients under 15 years of age [33]. Higher prevalence of *Actinomyces* in adults may be explained by poor oral hygiene and prolonged exposure to microinjuries of the oral and pharyngeal mucosa. The above factors promote colonization and penetration of *Actinomyces* into the tonsillar tissue [9,12,34].

We also investigated the prevalence of *Actinomyces* depending on sex. Our study found that tonsillar *Actinomyces* was present in 30.2% of women and 32.2% of men. However, the difference was not statistically significant. The results for this variable are in line with the results obtained by other authors [2,3,4,17,19,32,33,35].

The composition of the oral microbiome is influenced by many factors, including diet, body weight, and smoking habits. Considering the above, we decided to investigate the relationship between the occurrence of tonsillar *Actinomyces* and the above factors. We found that *Actinomyces* was more prevalent in the palatine tonsils of overweight or obese patients. To the best of our knowledge, no studies have assessed such a relationship. Furthermore, smoking did not seem to affect the prevalence of *Actinomyces*. In smokers, these bacteria were present in 37.7% of patients, while 36.9% of non-smokers presented the bacteria.

Much attention has been paid to the question of whether the presence of *Actinomyces* could lead to palatine tonsil hypertrophy [10,32]. The concept according to which tonsil colonization by *Actinomyces* could play an essential role in the hypertrophy of the tonsillar lymphoid tissue was first presented by Lord, who suggested that *Actinomyces* produced toxins that could lead to tonsillar hypertrophy [29,33,36]. However, it was not clearly confirmed. Many findings linking *Actinomyces* to tonsillar hypertrophy can be found in the literature. However, the mechanism that could result in tonsillar hypertrophy has not been clearly demonstrated yet. Many researchers have attempted to prove a link between the presence of *Actinomyces* and the hypertrophy of the pharyngeal lymphoid tissue [2,3,4,10,17,18,19,29,30,32,35].

Hari et al. found that 98% of patients with tonsillar *Actinomyces* presented with lymphoid hyperplasia compared to 77% without the bacteria. They emphasized that *Actinomyces* almost always led to tonsillar hyperplasia [29].

A significant effect of *Actinomyces* on tonsillar volume was demonstrated by Kutluhan and colleagues. The mean tonsillar volume was significantly higher in tonsils with *Actinomyces* than those without. *Actinomyces* was 5.71 times greater in hypertrophied tonsils than in recurrent tonsillitis. Kutluhan et al. uncovered a strong association between *Actinomyces* and tonsillar hypertrophy. However, the pathomechanism of these changes was not fully elucidated [3].

The role of *Actinomyces* in the etiology of tonsillar hypertrophy was also investigated by Daneshmandan and colleagues. The tonsil size was significantly greater in specimens positive for Actinomycosis compared to specimens negative for *Actinomyces* (8.65 ± 1.5 mL vs. 4.38 ± 0.22 mL, *p* < 0.001) [35]. Kansu also showed a statistically significant correlation between prevalence of *Actinomyces* and tonsils with higher volume [18]. Bhargava et al. showed a significant association between *Actinomyces* and tonsillar hypertrophy. Actinomycosis was present in 56.8% of patients with tonsillar hypertrophy compared to 10.3% of patients with recurrent tonsillitis without tonsillar hypertrophy [32]. According to Riffat and Walker, tonsillar hypertrophy associated with the presence of *Actinomyces* was related to lymphoid hyperplasia due to an antigen–antibody reaction or a cell-mediated phenomenon [23]. Pransky et al. also observed an association between tonsillar *Actinomyces* colonization and their hypertrophy [10]. 

In light of the above data, our results may be surprising. The mean tonsil size was smaller in the group of patients with *Actinomyces*. This difference was not statistically significant. However, these findings do not lead us to believe that *Actinomyces* causes tonsillar hypertrophy. Therefore, it can be suspected that the presence of tonsillar *Actinomyces* does not correlate with their size. Our results are different from the findings of many studies. Some authors, however, are of the opinion that the presence of tonsillar *Actinomyces* does not translate into tonsil increased size [2,4,17,19,30,37]. Our findings are in line with those obtained by Jones et al., who noted a negative correlation between tonsillar hypertrophy and tonsillar *Actinomyces* colonization. The authors indicated that these microorganisms should be regarded as saprophytes that do not affect the tonsil size [38].

In turn, a different perspective on this issue was presented by Yadav et al. in their case report. They found that *Actinomyces* resulted in massive unilateral tonsillar hypertrophy mimicking a tumor. Similarly, Kaipuzha et al. and Rasić et al. found that *Actinomyces* could cause unilateral tonsillar hypertrophy, thus causing tonsillar asymmetry [8,12,39].

Therefore, we also decided to investigate the relationship between the presence of *Actinomyces* and the occurrence of tonsillar asymmetry. However, the results did not confirm such a relationship.

Another broadly discussed issue is whether *Actinomyces* is more prevalent in patients with OSAS or chronic tonsillitis. In our study, *Actinomyces* was found in 43.4% of patients who underwent tonsillectomy for OSAS and in 29.1% of patients who underwent surgery for chronic tonsillitis. 

After excluding children from the analysis, the prevalence of *Actinomyces* in these two groups was 43% (OSAS) vs. 36.6% (chronic tonsillitis). The differences were not statistically significant. There are significant differences in data from many studies. The most similar results to our findings were obtained by Toh et al., who found *Actinomyces* in 44.1% of patients with OSAS and 33.3% of patients with recurrent tonsillitis [17]. As in our study, these differences were not statistically significant. 

Some studies found no significant association or differences in the prevalence of *Actinomyces* depending on the diagnosis (chronic tonsillitis vs. OSAS) [19,31,33,35]. Melgarejo et al. and Gaffney et al. stressed that *Actinomyces* should be considered a saprophyte that does not contribute to the above conditions [24,33]. In their histopathological study of tonsillectomy specimens, Sujatha et al. found that the presence of *Actinomyces* colonies was associated with the presence of a positive tissue reaction. They stressed that the etiological role of *Actinomyces* was significant in chronic tonsillitis, especially in antibiotic-resistant tonsillitis [14]. According to Daneshmandan et al., the presence of *Actinomyces* could be the reason for long-term problems with antibiotic-resistant tonsillar symptoms [35]. Ashraf et al. found a higher prevalence of *Actinomyces* colonies in patients with recurrent tonsillitis (43.9%) compared to those with OSAS (26.3%); however, the difference was not statistically significant. As opposed to Sujatha et al., Ashraf et al. found that the presence of *Actinomyces* in tonsil specimens did not indicate any active disease [4]. Gunizi et al. and Jones et al. also observed a higher prevalence of *Actinomyces* in patients who underwent surgery for recurrent palatine tonsillitis [30,38]. Additionally, many researchers showed that *Actinomyces* was found more commonly in specimens of tonsils removed due to OSAS than in those removed because of recurrent inflammation [3,10,17,20,22,23,32,37]. In a study on a large group of patients (n = 1213), Riffat and Walker found that *Actinomyces* was statistically more common in patients with obstructive symptoms [23]. These authors did not exclude its pathological role in the development of sleep-disordered breathing. According to them, one possible explanation for the difference between the sleep-disordered breathing group and the recurrent tonsillitis group was that children with recurrent tonsillitis had been often treated with multiple courses of broad-spectrum antibiotics and this could have an effect on the eradication of *Actinomyces*. They also showed that the involvement of *Actinomyces* in the pathogenesis of OSAS should be considered in further studies. They stressed that *Actinomyces* could not be dismissed as passive saprophytes [23].

Bhargava et al. expressed a similar opinion and suggested that *Actinomyces* could play a potential role in the development of obstructive tonsillar hypertrophy. In their study, *Actinomyces* was present in 56.8% of patients with OSAS and 10.3% of patients with recurrent tonsillitis [32]. In the study of Kutluhan et al., this ratio was 61.5% in patients with OSAS vs. 26.6% in those with recurrent tonsillitis. This difference was statistically significant (*p* < 0.001) [3]. In their study, Ozgursoy et al. also reported a higher prevalence of *Actinomyces* in patients undergoing tonsillectomy for obstructive symptoms. The presence of *Actinomyces* in tonsillectomy specimens did not indicate active tissue infection. They also suggested that *Actinomyces* could indirectly lead to OSAS by contributing to tonsillar hypertrophy [37].

The role of *Actinomyces* is also stressed in the context of the formation of tonsilloliths. Some studies showed that tonsillar cryptitis could be considered a histopathological indicator of Actinomycosis due to a significant correlation between the occurrence of *Actinomyces* and tonsillar cryptitis [12,31].

Arvisais-Anhalt et al. noted a significantly higher prevalence of *Actinomyces* in patients with tonsilloliths compared to those with recurrent tonsillitis. They emphasized that the prevalence of *Actinomyces* in tonsillolith tonsil specimens was high. However, they also stressed that *Actinomyces* routinely colonized non-tonsillolith tonsil specimens. Therefore, they questioned whether *Actinomyces* played an important role in the pathogenesis of this condition. They also reported that *Actinomyces*-targeted treatment of tonsilloliths failed to be effective [16].

In our study, no significant association was found between the occurrence of tonsilloliths and the presence of *Actinomyces* colonies. Due to the frequent coexistence of tonsilloliths and halitosis, our patients were also asked about the presence of halitosis. The analysis for the co-occurrence of *Actinomyces* and halitosis showed the result was close to statistical significance (*p* = 0.064). However, this finding did not clearly confirm that *Actinomyces* was responsible for the occurrence of this symptom.

Recently, more attention has been paid to studies on the role of the microbiome in the pathogenesis of some diseases. It is suspected that there is a link between microorganisms colonizing the upper respiratory tract and the development of asthma [32]. Zhao et al. determined that the core oropharyngeal microbiome of patients with asthma include *Prevotella*, *Streptococcus*, *Neisseria*, *Rothia*, *Haemophilus*, *Fusobacterium,* and *Actinomyces* [32]. Bhargava et al. found a statistically significant association (*p* < 0.0001) between *Actinomyces* in the palatine tonsils and bronchial asthma (*Actinomyces* was more prevalent in patients with asthma). However, those authors did not provide a hypothetical explanation for this relationship [32]. Our study found no significant relationship between the occurrence of bronchial asthma and *Actinomyces* in tonsillar tissue. 

We also analyzed the effect of the occurrence of *Actinomyces* on the postoperative period after bilateral tonsillectomy. An interesting retrospective study related to the occurrence of postoperative bleeding was conducted by Schrock et al., who analyzed 1522 patients who underwent palatine tonsillectomy. The group of patients in whom postoperative bleeding (7.7%) was found was compared with the group of patients without such a complication. The presence of tonsillar *Actinomyces* was significantly correlated with bleeding (*p* = 0.02). The odds ratio, however, was low (OR 2.0). Despite a significant correlation between Actinomycosis and postoperative bleeding, Schrock et al. showed that *Actinomyces* only slightly increased the risk of postoperative bleeding. Further studies are needed to determine the importance of other contributing factors [32]. 

The above study was the stimulus which prompted our investigation into these relationships in our patient population. The analysis was based on the data obtained from 100 patients who were asked about the occurrence of postoperative bleeding. This complication occurred in 12 patients. The estimated probability of postoperative bleeding was not significantly associated with the presence of *Actinomyces* in histopathological specimens. We also assessed the influence of *Actinomyces* on the need for empirical postoperative antibiotic therapy due to surgical site infection. 

Based on the analyses, complications in the form of surgical site infection were not associated with the presence of *Actinomyces* in the postoperative specimens. To the best of our knowledge, a study evaluating similar variables has yet to been conducted. Our study analyzed the effect of *Actinomyces* on the severity of postoperative pain. It is known that inflammation affects the sensory innervation of tissues and organs. Therefore, we decided to assess this relationship. Patients usually describe tonsillectomy-related pain as a pain of significant severity, which significantly reduces the quality of life in the postoperative period [40].

Among the respondents, the largest number of patients (45.5%) reported severe pain (VAS 7–9). However, no significant differences were noted in the severity of pain after tonsillectomy depending on the presence of *Actinomyces* in the postoperative specimens. The presence of tonsillar *Actinomyces* does not seem to indicate active inflammation in the oropharyngeal tissues, which could translate into the course of the postoperative period. Zagólski et al. evaluated postoperative pain according to the indications for tonsillectomy. They assessed the effect of recurrent inflammation on pain severity. Patients with recurrent tonsillitis reported less severe postoperative pain than patients undergoing tonsillectomy due to other indications. Higher pain intensity in patients without a history of recurrent tonsillitis was explained by the fact that they had not expected such a severe pain. Patients with recurrent tonsillitis presented with a history of disabling sore throat episodes [40].

## 5. Conclusions

The hypothesis that *Actinomyces* plays an essential role in the development of the pathology of the palatine tonsils was not confirmed. The presence of the bacteria in the postoperative specimens without other concomitant clinical features should be regarded as a fortuitous finding which is not related to active disease. We did not find a significant correlation between tonsillar *Actinomyces* colonization and the conditions that were assessed. However, the result was close to statistical significance for the co-occurrence of *Actinomyces* and halitosis. Age was the only statistically significant variable because it affected the prevalence of tonsillar *Actinomyces* colonies, a phenomenon which is in line with the reports from most authors. In addition, these tonsillar bacteria did not affect the postoperative period. The multivariate analysis showed that *Actinomyces* should be considered saprophytes of the oropharyngeal microflora that are not related to the occurrence of pathology of the palatine tonsils. However, further studies are warranted to assess the influence of the bacteria on the occurrence of halitosis to provide a conclusive answer about the role of *Actinomyces* in the occurrence of this symptom.

## Figures and Tables

**Table 1:** Age for patients with and without *Actinomyces*.

*Actinomyces*	M	SD	Me	IQR	Min	Max	n	Sk
No	34.74	11.39	34	15	18	66	242	0.61
Yes	38.21	13.4	37	18.5	18	82	147	0.68

M—mean, SD—standard deviation, Me—median, IQR—interquartile range, Min—minimum value, Max—maximum value, n—group size, Sk—skewness.

**Table 2:** Welch’s t-test results for the size of palatine tonsils in the patient groups (A+ and A−).

*Actinomyces*	M	SD	LI	UI	sk	N	t	df	P	d
A−	2.39	0.66	2.32	2.47	−0.06	286	1.94	304.12	0.053	0.19
A+	2.27	0.55	2.18	2.37	0.3	133				

M—mean, SD—standard deviation, LI and UI—lower and upper confidence intervals, sk—skewness of the distribution, n—group size, t—the value of the t statistic, df—degrees of freedom, *p*—the value of the *p* statistic, d—the value of Cohen’s d effect size.

## Data Availability

The data used to support the findings of this research are available upon request.
